# Scheduled QR-BP Detector with Interference Cancellation and Candidate Constraints for MIMO Systems

**DOI:** 10.3390/s21113734

**Published:** 2021-05-27

**Authors:** Sangjoon Park

**Affiliations:** Department of Electronic Engineering, Kyonggi University, Suwon 16227, Korea; sj.park@kgu.ac.kr

**Keywords:** belief propagation, MIMO systems, QR decomposition, interference cancellation, candidate constraint

## Abstract

In this paper, a QR-decomposition-based scheduled belief propagation (BP) detector with interference cancellation (IC) and candidate constraints is proposed for multiple-input multiple-output (MIMO) systems. Based on a bipartite graph generated from an upper triangular channel matrix following linear transformation using QR decomposition, the proposed detector performs a sequential message updating procedure between bit nodes. During this updating procedure, candidate constraints are imposed to restrict the number of possible candidate vectors for the calculation of observation-to-bit messages. In addition, after obtaining the soft message corresponding to the bit sequence in each transmit symbol, a hard-decision IC operation is performed to reduce the size of the bipartite graph and indirectly update the messages for the remaining symbols. Therefore, the proposed scheme provides a huge complexity reduction compared to conventional BP detectors that perform message updating by using all related messages directly. Simulation results confirm that the proposed detector can achieve suboptimum error performance with significantly improved convergence speed and reduced computational complexity compared to conventional BP detectors in MIMO systems.

## 1. Introduction

In multiple-input multiple-output (MIMO) systems, signals are simultaneously sent from multiple transmit antennas, and a receiver must estimate and detect the transmitted signals [1,2]. Although the linear detection schemes, such as linear zero-forcing (LZF) and linear minimum mean-square-error (LMMSE), can perform simple detection procedures with low complexity, their accuracy is relatively poor. In contrast, optimal detection schemes, such as maximum likelihood (ML), incur massive computational complexity that increases exponentially with the number of transmit antennas. Therefore, many alternative detection schemes have been investigated for MIMO systems.

Message passing (MP) algorithms are known to solve a wide variety of problems by factorizing the global function of variables into a combination of simpler local functions [3,4,5]. Among MP approaches, belief propagation (BP) algorithms perform inference on graphical models (e.g., bipartite graphs) [6], and they have been widely applied for channel decoding problems [7,8] and detection problems [9,10,11,12,13,14,15]. These BP algorithms can achieve suboptimum performance, and thereby a number of BP detectors have been developed for MIMO systems.

In a BP detector, messages from bit and observation nodes are iteratively exchanged based on a bipartite graph that is utilized for detection. However, the bipartite graph for a basic MIMO system model is fully connected, that is, every bit node (observation node) is connected to all observation nodes (bit nodes) by edges, where transmitted bits and received signals are interpreted as bit and observation nodes, respectively. Therefore, based on large numbers of nodes and edges, a BP detector based on a fully connected bipartite graph for MIMO systems (referred to as the standard BP detector hereafter) incurs huge computational complexity that can even exceed that of ML detection [10]. Therefore, several methods for reducing the computational complexity of the standard BP detector have been studied.

One of the simplest approaches is the edge pruning technique [10,11]. In edge pruning, the calculation of messages for selected edges is forcefully omitted in each BP iteration, where the selection is performed based on a given criterion, e.g., channel gain. Although significant reductions in complexity can be achieved, error performance is also reduced compared to the standard BP detector. As another low complexity approach, in [14], a BP detector with damping on the a priori probability was developed, although it is designed for large-scale MIMO systems with spatial correlation. In addition, in [15], a BP-based deep learning detector for low complexity detection was investigated for MIMO systems, which requires additional overhead for training.

For general MIMO systems without incurring extra overhead, QR-decomposition-based BP detectors (referred to as QR-BP detectors hereafter) are known to show a lower complexity and better error performance compared with the standard BP detector [12,13]. In QR-BP detectors, QR decomposition of the MIMO channel matrix is performed, and the unitary matrix obtained from QR decomposition is used for the linear transformation of the basic MIMO system model. Based on the upper triangular structure of the channel matrix following linear transformation, the bipartite graph for the linearly transformed channel matrix contains a smaller number of edges than the fully connected bipartite graph, which reduces the number of calculated messages. In addition, the number of cycles in the bipartite graph is also reduced compared to the fully connected bipartite graph without information loss on the likelihoods estimated from noisy observations via linear transformation using a unitary matrix [16,17]. Therefore, QR-BP detectors can obtain better performance with less computational complexity compared to the standard BP detector. However, QR-BP detectors still incur huge computational complexity that increases exponentially with the number of transmit antennas. Furthermore, standard BP and QR-BP detectors employ parallel message updating procedures, that is, all messages in the current BP iteration are updated simultaneously using messages obtained from the previous BP iteration. Therefore, these methods require many BP iterations for convergence, which increases complexity in practical applications further.

Therefore, to obtain additional reductions in computational complexity while accelerating convergence speed and minimizing performance loss, this paper proposes a scheduled QR-BP detector with interference cancellation (IC) and candidate constraints (referred to as the QR-SBP detector hereafter) for MIMO systems. The aim of this study can be summarized as below:

- The proposed QR-SBP detector is based on the bipartite graph from the upper triangular matrix obtained by the linear transformation using QR decomposition. Therefore, similar to QR-BP detectors, the numbers of edges and cycles are reduced compared to the fully connected graph for the standard BP detector.
- In order to accelerate the convergence speed from QR-BP detectors, the proposed QR-SBP detector performs sequential updating of bit-to-observation and observation-to-bit messages for each transmit symbol, which is motivated by scheduled BP decoding algorithms for channel codes [7,8]. Therefore, the messages for the last transmit symbol updated in the current BP iteration are utilized for updating the messages for the next transmit symbol, which can significantly accelerate convergence.
- Furthermore, for a smaller complexity compared to other BP detectors, the hard-decision IC operation is applied to the effective receive signal vector by regenerating the transmit symbols using the updated messages. Then, the sizes of the effective system model and corresponding bipartite graph are reduced after IC. Therefore, unlike existing BP detectors that calculate messages directly from all related messages, the proposed scheme can reduce the number of other messages required for updating each message, which reduces overall computational complexity.
- In addition, for additional complexity reductions, the proposed QR-SBP detector employs a candidate constraint strategy during the updating of observation-to-bit messages for a given transmit symbol. Specifically, the number of candidate vectors used for updating observation-to-bit messages is limited, which facilitates additional complexity reduction.

As a result, the computational complexity of the proposed QR-SBP detector is approximately proportional to the square of the number of transmit antennas, where the standard BP and QR-BP detectors have complexity that increases exponentially with the number of transmit antennas. Therefore, compared with the conventional BP detectors, the proposed QR-SBP detector yields a significantly accelerated convergence speed and a huge complexity reduction.

The remainder of the paper is organized as follows. Section 2 describes the MIMO system model. Section 3 introduces the conventional standard BP and QR-BP detectors. Section 4 presents the detailed procedures of the proposed QR-SBP detector. Section 5 presents simulation results that verify the performance of the proposed scheme. Finally, Section 6 concludes this paper.

**N**otation. *Throughout the paper, the following notations are used. Lower-case and upper-case boldface letters denote vectors and matrices, respectively. The superscripts T and H denote the transpose and transpose-and-conjugate operators, respectively. [·,·] and [·;·] denote column-wise and row-wise aggregation of elements, respectively. A(:,a:b) denotes a submatrix of A containing its ath to bth columns and A(a:b,:) denotes a submatrix of A containing its ath to bth rows. $0_{a\times b}$ and $I_{a}$ denote the a×b all-zero matrix and a×a identity matrix, respectively. E[·] denotes the expectation operation and ⌈·⌉ denotes the ceiling operation.*

## 2. System Model

We consider a spatially multiplexed MIMO system in which the numbers of transmit and receive antennas are *K* and *M*(≥*K*), respectively. This can represent both single-user MIMO (e.g., a point-to-point M×K MIMO system) and multi-user MIMO (e.g., an uplink MIMO system with *K* single-antenna users and *M* antennas at the base station) systems. Let $\left\{b_{j,1},\cdots ,b_{j,KQ}\right\}$ denote the bit sequence for the *j*th transmit signal vector in the current transmission time slot, where $\left\{b_{j,(k-1)Q+1},\cdots ,b_{j,kQ}\right\}$ is the bit sequence sent from the k(1≤k≤K)th transmit antenna. *Q* is the number of bits assigned for each transmit symbol (i.e., the modulation order is $2^{Q}$). For modulation, we consider a $2^{Q}$-ary constellation set X that satisfies $\sum_{x\in X}x=0$ and $\sum_{x\in X}{\left|x\right|}^{2}/2^{Q}=1$.

Without loss of generality, the index *j* is omitted for notational simplicity. Then, $\left\{b_{1},\cdots ,b_{KQ}\right\}$ denotes the bit sequence in the current transmission time slot, where $\left\{b_{(k-1)Q+1},\cdots ,b_{kQ}\right\}$ represents the bit sequence sent from the *k*th transmit antenna and N=KQ is the length of the bit sequence in each transmit signal vector. Let $x={\left[x_{1},\cdots ,x_{K}\right]}^{T}$ be a K×1 transmit signal vector for the current transmission time slot, where each $x_{k}\left(1\leq k\leq K\right)$ generated from $\left\{b_{(k-1)Q+1},\cdots ,b_{kQ}\right\}$ is the transmit symbol at the *k*th transmit antenna. Then, according to the general input-output relationship of a spatially multiplexed MIMO system [1,2,3,4,9,10,11,12,13,14,15], the M×1 receive signal vector $y={\left[y_{1},\cdots ,y_{M}\right]}^{T}$ can be written as

(1) y=Hx+n.

In (1), H is an M×K full-rank MIMO fading channel matrix (i.e., rank(H)=K), where E[|H(m,k)|]=1 for 1≤m≤M and 1≤k≤K, and n is an M×1 additive white Gaussian noise (AWGN) vector whose elements are independent and identically distributed complex Gaussian random variables with zero mean and variance $\sigma^{2}$. Because each $x_{k}$ is a member of X, $E\left[xx^{H}\right]=I_{K}$.

## 3. Conventional BP Detectors

In this section, the detailed procedures of the conventional BP detectors are described. Two BP detectors closely related to the proposed QR-SBP detector, the standard BP detector [10] and QR-BP detector [12] are introduced.

### 3.1. Standard BP Detector

In the standard BP detector, a conventional MIMO channel matrix H is modeled as a bipartite graph containing *M* observation nodes (corresponding to the received symbols $\left\{y_{1},\cdots ,y_{M}\right\}$) and *N*(=KQ) bit nodes (corresponding to the bit sequence $\left\{b_{1},\cdots ,b_{N}\right\}$). Soft messages are iteratively generated and exchanged between the bit and observation nodes. Based on the use of H, the bipartite graph for the standard BP detector is fully connected. Therefore, let $\alpha_{nm}$ for 1≤n≤N and 1≤m≤M denote the message sent from the *n*th bit node to the *m*th observation node, and let $\beta_{mn}$ for 1≤m≤M and 1≤n≤N denote the message sent from the *m*th observation node to the *n*th bit node. In addition, let $X^{K,b}$ denote the set of all possible K×1 vectors whose elements are the members of the constellation set X with $b_{n}=b$ for b=0 and 1. Then, by using the max-log approximation of $\log\left(e^{x}+e^{y}\right)\approx \max\left(x,y\right)$, each $\beta_{mn}$ for 1≤m≤M and 1≤n≤N can be calculated as [10]

(2) $$\begin{array}{c} \beta_{mn}\approx \max_{x^{\prime }\in X^{K,1}}\left\{-|y_{m}-h_{m}x^{\prime }{|}^{2}+\sum_{i=1:b_{i}=1,i\neq n}^{N}\alpha_{im}\right\}\\ -\max_{x^{\prime }\in X^{K,0}}\left\{-|y_{m}-h_{m}x^{\prime }{|}^{2}+\sum_{i=1:b_{i}=1,i\neq n}^{N}\alpha_{im}\right\}, \end{array}$$

where $h_{m}$ is a 1×K row vector corresponding to the *m*th row of H and $\alpha_{im}$ is the most recently generated message from the *i*th bit node to the *m*th observation node. Note that all $\alpha_{nm}$ should be initialized to zero before the beginning of the first BP iteration.

After obtaining $\beta_{mn}$, $\alpha_{nm}$ for 1≤n≤N and 1≤m≤M can be simply updated as

(3) $$\alpha_{nm}=\sum_{i=1;i\neq m}^{M}\beta_{in}.$$

Equations (2) and (3) are performed repeatedly until a stopping criterion is satisfied (e.g., the maximum number of BP iterations). If the soft output of the *n*th bit $b_{n}$ is required after the *t*th BP iteration, then the log-likelihood ratio (LLR) $l_{n}$ can be obtained as

(4) $$l_{n}=\sum_{i=1}^{M}\beta_{in}=\alpha_{nm}+\beta_{mn}\;\forall m.$$

By exchanging $\alpha_{nm}$ and $\beta_{mn}$ in an iterative manner, the standard BP detector can obtain fine error performance if a sufficient number of BP iterations are performed. However, the cardinality of $X^{K,b}$ in (2) is $2^{KQ-1}$(=$2^{N-1})$ for both b=0 and 1. Therefore, the computational complexity of the standard BP detector for each iteration exponentially increases with *K* and *Q*. In addition, because the fully connected bipartite graph is a loopy graph containing many cycles, the convergence of the standard BP detector is not guaranteed [18]. Therefore, the standard BP detector requires a number of BP iterations to achieve acceptable error performance, which is impractical when considering the huge computational load of each BP iteration.

### 3.2. QR-BP Detector

In the QR-BP detector, QR decomposition is performed on the original channel matrix H and linear transformation is performed using the unitary matrix obtained from QR decomposition. Specifically, following QR decomposition, H can be rewritten as

(5) $$H=Q[R;0_{(M-K)\times K}],$$

where Q is an M×M unitary matrix and $R=[r_{1};\cdots ;r_{K}]$ is a K×K upper triangular matrix with a 1×K row vector $r_{m}$ for 1≤m≤K, where the first (m−1) elements in $r_{m}$ are equal to zero (i.e., $r_{m}=\left[0_{1\times (m-1)},r_{m,m},\cdots ,r_{m,K}\right]$). Because $\left[I_{K},0_{K\times (M-K)}\right]Q^{H}H=R$, based on the multiplication of $\left[I_{K},0_{K\times (M-K)}\right]Q^{H}$, y in (1) can be transformed as

(6) $$\tilde{y}=\left[I_{K},0_{K\times (M-K)}\right]Q^{H}y=\mathrm{Rx}+\tilde{n},$$

where $\tilde{y}={\left[{\tilde{y}}_{1},\cdots ,{\tilde{y}}_{K}\right]}^{T}$ and $\tilde{n}=\left[I_{K},0_{K\times (M-K)}\right]Q^{H}n$ are the K×1 receive signal and AWGN vectors following linear transformation, respectively, and the new channel matrix R can be written as

(7) $$R=\left[\begin{array}{c} r_{1}\\ r_{2}\\ \vdots \\ r_{K} \end{array}\right]=\left[\begin{array}{cccc} r_{1,1} & r_{1,2} & \cdots & r_{1,K}\\ 0 & r_{2,2} & \cdots & r_{2,K}\\ \vdots & \ddots & \ddots & \vdots \\ 0 & \cdots & 0 & r_{K,K} \end{array}\right].$$

Therefore, after linear transformation based on QR decomposition, the original M×K MIMO system model in (1) with H is represented by the K×K MIMO system model in (6) with R. Because of the upper triangular structure of R, the bipartite graph based on (6) is not fully connected. Therefore, the numbers of $\alpha_{nm}$ and $\beta_{mn}$ are reduced compared to the standard BP detector. Specifically, corresponding to the non-zero elements of R, $\beta_{mn}$ exists for 1≤m≤K and (m−1)Q+1≤n≤N, and $\alpha_{nm}$ exists for 1≤n≤N and $1\leq m\leq \left\lceil n/Q\right\rceil$. Then, in the *t*th BP iteration, each $\beta_{mn}$ is given by [12]

(8) $$\begin{array}{c} \beta_{mn}\approx \max_{x^{\prime }\in X^{K-m+1,1}}\left\{\gamma_{m}\left(x^{\prime }\right)\;+\;\sum_{i=\left(m\;-\;1\right)Q+1:b_{i}=1,i\neq n}^{N}\alpha_{im}\right\}\\ -\max_{x^{\prime }\in X^{K-m+1,0}}\left\{\gamma_{m}\left(x^{\prime }\right)\;+\;\sum_{i=\left(m\;-\;1\right)Q+1:b_{i}=1,i\neq n}^{N}\alpha_{im}\right\}, \end{array}$$

where $\gamma_{m}\left(x^{\prime }\right)=-|{\tilde{y}}_{m}-{\tilde{r}}_{m}x^{\prime }{|}^{2}$ and ${\tilde{r}}_{m}$ is a 1×(K−m+1) vector containing the non-zero elements of $r_{m}$. In addition, $X^{a,b}$ for b=0 and 1 in (8) denotes the set of all the possible a×1 vectors whose elements are members of the constellation set X with $b_{n}=b$. Note that the entire $\alpha_{nm}$ for 1≤n≤N and $1\leq m\leq \left\lceil n/Q\right\rceil$ should be initialized to zero prior to the first BP iteration, as in the standard BP detector.

Following the calculation of $\beta_{mn}$, $\alpha_{mn}$ can be obtained as

(9) $$\alpha_{nm}=\sum_{i=1;i\neq m}^{\left\lceil n/Q\right\rceil }\beta_{in}.$$

Similar to the standard BP detector, (8) and (9) are applied repeatedly for the QR-BP detector until a stopping criterion is satisfied. After the *t*th BP iteration, the soft output of the *n*th bit $b_{n}$, $l_{n}$, can be obtained as

(10) $$l_{n}=\sum_{i=1}^{\left\lceil n/Q\right\rceil }\beta_{in}=\alpha_{nm}+\beta_{mn}\;\forall m\leq \left\lceil n/Q\right\rceil .$$

Therefore, instead using a fully connected bipartite graph based on H as in the standard BP detector, a bipartite graph from an upper triangular matrix R is utilized in the QR-BP detector based on QR decomposition and linear transformation. No information is lost by using the unitary matrix $Q^{H}$ for the linear transformation [16,17]. Consequently, by having fewer edges and cycles, the QR-BP detector can provide improved error performance with reduced computational complexity per BP iteration compared to the standard BP detector. However, because $\beta_{mn}$ is still updated in parallel using $\alpha_{im}$ generated from the last BP iteration as in (8), convergence speed is not significantly accelerated compared to the standard BP detector [12]. Furthermore, because the maximum cardinality of $X^{K-m+1,b}$ in (8) is still $2^{KQ-1}\left(=2^{N-1}\right)$ when m=1, the computational complexity of the QR-BP detector still increases exponentially with *K* and *Q*.

## 4. Proposed QR-SBP Detector with IC and Candidate Constraints

**Figure 1:**
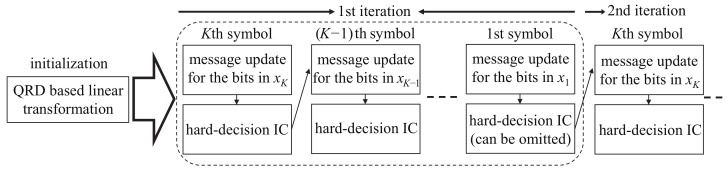
Illustration of the overall sequential procedure of the proposed QR-SBP detector.

In this section, the details of the proposed QR-SBP detector are described, where the overall sequential procedure is illustrated in Figure 1. In the proposed QR-SBP detector, QR decomposition and the corresponding linear transformation are performed first, as in (5) to (7) for the QR-BP detector, before beginning the detection process. Therefore, a bipartite graph from the upper triangular matrix R is used instead of a fully connected graph from the original channel matrix H. Furthermore, as in the standard and QR-BP detectors, all bit-to-observation messages $\alpha_{nm}$ are initialized to zero prior to the beginning of the detection procedure.

In each BP iteration, the proposed QR-SBP detector performs a bit-by-bit message updating procedure from the last transmit symbol ($x_{K}$) to the first transmit symbol ($x_{1}$). In other words, during each BP iteration, the message updating procedure from $x_{K}$ to $x_{1}$ is performed sequentially for the bit sequence included in each transmit symbol, i.e., q=1 to q=Q for $b_{(K-1)Q+q}$ in $x_{K}$, q=1 to q=Q for $b_{(K-2)Q+q}$ in $x_{K-1}$, etc. At the end of the message updating procedure for a bit sequence in $x_{k+1}$, a hard decision symbol ${\hat{x}}_{k+1}$ is generated and canceled based on the receive signal vector. Let ${\tilde{y}}^{\left(k\right)}$ denote the receive signal vector used for the message updating of the bit sequence in $x_{k}$ after the hard-decision IC of ${\hat{x}}_{k+1}$, where ${\tilde{y}}^{\left(K\right)}=\tilde{y}$ because there is no IC operation. Then, ${\tilde{y}}^{\left(k\right)}$ can be represented as

(11) $${\tilde{y}}^{\left(k\right)}={\tilde{y}}^{\left(k+1\right)}-r_{k}{\hat{x}}_{k}.$$

Because of hard-decision IC operations, the effects of the symbols $\left\{x_{k+1},\cdots ,x_{K}\right\}$ do not need to be considered for the message updating procedures of $\left\{b_{(k-1)Q+1},\cdots ,b_{kQ}\right\}$ in $x_{k}$. Specifically, if the $k^{\prime }\left(k+1\leq k^{\prime }\leq K\right)$th column of the linearly transformed channel matrix R is eliminated by the cancellation of $x_{k^{\prime }}$, then the $m^{\prime }\left(k+1\leq m^{\prime }\leq K\right)$th row of the resulting R(:,1:k) channel matrix becomes a 1×k all-zero vector by the upper triangular structure of R. Consequently, the $m^{\prime }$th row of R(:,1:k) also does not need to be considered during message calculation. Therefore, following the hard-decision IC operations of $\left\{x_{k+1},\cdots ,x_{K}\right\}$, the system model in (6) can be reformulated for the message updating of the bit sequence in $x_{k}$ as

(12) $$z^{\left(k\right)}=R^{\left(k\right)}x^{\left(k\right)}+{\tilde{n}}^{\left(k\right)},$$

where $z^{\left(k\right)}={\tilde{y}}^{\left(k\right)}\left(1:k\right)={\left[z_{1}^{\left(k\right)},\cdots ,z_{k}^{\left(k\right)}\right]}^{T}$ is a k×1 effective receive signal vector with $z^{\left(K\right)}=\tilde{y}$, $x^{\left(k\right)}=x\left(1:k\right)={\left[x_{1},\cdots ,x_{k}\right]}^{T}$ is a k×1 effective transmit signal vector, ${\tilde{n}}^{\left(k\right)}=\tilde{n}\left(1:k\right)$ is a k×1 effective AWGN vector, and $R^{\left(k\right)}=R\left(1:k,1:k\right)$ is a k×k effective channel matrix defined as

(13) $$R^{\left(k\right)}=\left[\begin{array}{c} r_{1}\left(1:k\right)\\ r_{2}\left(1:k\right)\\ \vdots \\ r_{k}\left(1:k\right) \end{array}\right]=\left[\begin{array}{cccc} r_{1,1} & r_{1,2} & \cdots & r_{1,k}\\ 0 & r_{2,2} & \cdots & r_{2,k}\\ \vdots & \ddots & \ddots & \vdots \\ 0 & \cdots & 0 & r_{k,k} \end{array}\right].$$

Therefore, by performing IC operations, a smaller system model can be used as *k* decreases, which leads to complexity reduction during the message updating procedure based on a smaller bipartite graph.

After the hard-decision IC operations using ${\hat{x}}_{k+1}\left(2\leq k+1\leq K\right)$, the message updating procedure for $b_{(k-1)Q+q}$ in $x_{k}$ is performed sequentially from q=1 to *Q*, where the update order of the bit sequence in each symbol can be determined arbitrarily. Then, for $b_{(k-1)Q+q}$, the observation-to-bit message $\beta_{mn}$ with n=(k−1)Q+q is sequentially calculated from each $z_{m}^{\left(k\right)}$. The sequential updating order of $\beta_{mn}$ for a given *n* is from m=k to m=1 because an observation node with a larger *m* is connected to a smaller number of bit nodes in the bipartite graph based on the upper triangular matrix $R^{\left(k\right)}$. When m=k, then $\beta_{mn}$ with n=(k−1)Q+q is initially obtained as

(14) $$\begin{array}{c} \beta_{mn}\approx \max B_{mn}^{1}-\max B_{mn}^{0}, \end{array}$$

where the set $B_{mn}^{b}$ with b=0 and 1 is defined as

(15) $$\begin{array}{c} \begin{array}{c} B_{mn}^{b}\;=\;\left\{\left.\gamma_{m}^{\left(k\right)}\left(x^{\prime }\right)\;+\;\sum_{i=\left(m\;-\;1\right)Q\;+\;1:b_{i}=1,i\neq n}^{kQ}\alpha_{im}\right|x^{\prime }\;\in \;X_{\left(k\right)}^{w,b}\right\} \end{array}. \end{array}$$

In (15), w=k−m+1, $\gamma_{m}^{\left(k\right)}\left(x^{\prime }\right)=-|z_{m}^{\left(k\right)}-{{\tilde{r}}_{m}}^{\left(k\right)}x^{\prime }{|}^{2}$, ${{\tilde{r}}_{m}}^{\left(k\right)}$ is a 1×w vector containing the non-zero elements of the *m*th row of $R^{\left(k\right)}$, and $X_{\left(k\right)}^{w,b}$ is a set of the w×1 vectors whose elements are the members of X with $b_{n}=b$.

To reduce computational complexity further for the calculation of (14) and (15), in addition to the hard-decision IC operation, a candidate constraint strategy is employed in the proposed QR-SBP detector. Specifically, the cardinality of $X_{\left(k\right)}^{w,b}$, $|X_{\left(k\right)}^{w,b}|$ is limited for complexity reduction. Let $\delta_{\left(k\right)}^{w}$ denote the number of surviving w×1 vectors that should remain after the calculation of $\beta_{mn}$ for the next observation-to-bit message $\beta_{(m-1)n}$. Then, after the calculation of $\beta_{mn}$ in (14), the $\delta_{\left(k\right)}^{w}$ elements in $X_{\left(k\right)}^{w,b}$ with the largest values in $B_{mn}^{b}$ are retained and selected to reduce the number of candidates considered for the next observation-to-bit message updating procedure. Let $Y_{\left(k\right)}^{w,b}$ denote the set containing the $\delta_{\left(k\right)}^{w}$ surviving w×1 vectors in $X_{\left(k\right)}^{w,b}$. Then, by initially setting $Y_{\left(k\right)}^{w,b}=\emptyset$, the following procedure is repeated $\delta_{\left(k\right)}^{w}$ times to select the $\delta_{\left(k\right)}^{w}$ surviving vectors in $X_{\left(k\right)}^{w,b}$:

(16) $$\begin{array}{c} Y_{\left(k\right)}^{w,b}=Y_{\left(k\right)}^{w,b}\cup \underset{x^{\prime }\in X_{\left(k\right)}^{w,b}\backslash Y_{\left(k\right)}^{w,b}}{\arg\max}B_{mn}^{b} \end{array}.$$

Then, the set $X_{\left(k\right)}^{w+1,b}$, which is used for the next observation-to-bit message $\beta_{(m-1)n}$, is obtained using the surviving vectors in $Y_{\left(k\right)}^{w,b}$ as

(17) $$X_{\left(k\right)}^{w+1,b}=\left\{\left.\left[\begin{array}{c} x^{\prime }\\ y^{\prime } \end{array}\right]\right|x^{\prime }\in X,y^{\prime }\in Y_{\left(k\right)}^{w,b}\right\}.$$

Consequently, because $|Y_{\left(k\right)}^{w,b}|=\delta_{\left(k\right)}^{w}$, the cardinality of $X_{\left(k\right)}^{w+1,b}$ is limited to $|X_{\left(k\right)}^{w+1,b}|=2^{Q}\cdot \delta_{\left(k\right)}^{w}$. In this manner, the candidates for observation-to-bit message updating are constrained by the surviving vector selection after IC, which reduces the complexity of the proposed scheme. Equations (16) and (17) do not need to be performed for the final $\beta_{mn}$ with m=1(w=k) because there are no remaining observation nodes to be considered for the updating of $\beta_{1n}$. Furthermore, the cardinality of $X_{\left(k\right)}^{1,b}$ (i.e., the first candidate set used to obtain $\beta_{mn}$ with m=k(w=1) for $b_{n}$) is limited to $2^{Q-1}$ because there are $2^{Q-1}$ symbols in X for a given $b(=b_{n})$. Therefore, $1\leq \delta_{\left(k\right)}^{1}\leq 2^{Q-1}$ and $1\leq \delta_{\left(k\right)}^{w}\leq 2^{Q}$ for 2≤w≤k−1. Note that a higher $\delta_{\left(k\right)}^{w}$ can improve detection performance by including a larger number of candidate vectors, whereas a smaller $\delta_{\left(k\right)}^{w}$ can reduce complexity for the calculation of $\beta_{mn}$, which is the main complexity burden of BP detectors.

The procedures in (14) to (17) are performed repeatedly from m=k to m=1 (w=1 to w=k) to obtain all $\beta_{mn}$ for $b_{n(=(k-1)Q+q)}$. After obtaining the entire $\beta_{mn}$ from m=k to m=1 for $b_{n}$, the bit-to-observation message $\alpha_{nm}$ can be calculated. Considering the k×k effective channel matrix $R^{\left(k\right)}$, $\alpha_{nm}$ is calculated for n=(k−1)Q+q and 1≤m≤k as

(18) $$\alpha_{nm}=\sum_{i=1;i\neq m}^{k}\beta_{in},$$

which is the end of the message updating procedures for $b_{(k-1)Q+q}$. The procedures in (14) to (18) are sequentially performed from q=1 to q=Q for $\left\{b_{(k-1)Q+1},\cdots ,b_{kQ}\right\}$ in $x_{k}$.

After obtaining all $\alpha_{nm}$ and $\beta_{mn}$ for $\left\{b_{(k-1)Q+1},\cdots ,b_{kQ}\right\}$ in $x_{k}$, $l_{n}$ for (k−1)Q+1≤n≤kQ can be calculated as

(19) $$l_{n}=\sum_{i=1}^{k}\beta_{in}=\alpha_{nm}+\beta_{mn}\;\forall \;1\leq m\leq k.$$

The hard-decision symbol ${\hat{x}}_{k}$ can then be generated after obtaining all soft messages for $\left\{b_{(k-1)Q+1},\cdots ,b_{kQ}\right\}$, and ${\hat{x}}_{k}$ is used in the IC operation of $x_{k}$ for the bits in the remaining symbols $\left\{x_{1},\cdots ,x_{k-1}\right\}$ in the current BP iteration.

To sum up, the message updating process of the proposed QR-SBP detector can be described as follows:

**(0)**  (Initialization) Perform QR decomposition on H using (5) and linear transformation using (6). Further, set t:=1, all $\alpha_{nm}$ to zero, and k:=K. Finally, set $\delta_{w}^{\left(k^{\prime }\right)}$ for $1\leq k^{\prime }\leq K$ and $1\leq w\leq k^{\prime }$.
**(1)**  (Preparation for calculating messages for the bit sequence in $x_{k}$) Set q:=1 and n:=(k−1)Q+1.
  **(1.1)**  (Initialization for $b_{n}$) Set m:=k. In addition, set $X_{\left(k\right)}^{1,0}$ and $X_{\left(k\right)}^{1,1}$ using X.
    **(1.1.1)**  (Update observation-to-bit messages) Update $\beta_{mn}$ using (14) and (15).
    **(1.1.2)**  (Select surviving vectors) If m>1, calculate $X_{\left(k\right)}^{k-(m-1)+1,b}$ for b∈{0,1} using (16) and (17).
    **(1.1.3)**  (Check remaining observation nodes for a given bit node) Set m:=m−1. If m≥1, then go back to **(1.1.1)**. Otherwise, go to **(1.1.4)**.
    **(1.1.4)**  (Update bit-to-observation messages) Update all $\alpha_{nm}$ for 1≤m≤k using (18).
  **(1.2)**  (Check remaining bit nodes for a given transmit symbol) Set q:=q+1 and n:=n+1. If q≤Q, then go back to **(1.1)**. Otherwise, go to **(2)**.
**(2)**  (LLR calculation and hard-decision symbol generation) Calculate $l_{n}$ for (k−1)Q+1≤n≤kQ using (19) and generate ${\hat{x}}_{k}$.
**(3)**  (Hard-decision IC and system model reformulation) If k>1, perform hard-decision IC with $\hat{x_{k}}$ and system model reformulation using (11) to (13).
**(4)**  (Check remaining transmit symbols) Set k:=k−1. If k≥1, then go back to **(1)**. Otherwise, go to **(5)**.
**(5)**  (Check iteration stopping criterion(s)) If an additional iteration is required, then set t:=t+1, k:=K, and go back to **(1)**.

The main characteristics of the proposed QR-SBP detector can be summarized as follows:

- The proposed QR-SBP detector is based on a linearly transformed system model using QR decomposition, which yields smaller numbers of edges and cycles in the bipartite graph compared to the fully connected bipartite graph used in the original system model.
- By using candidate constraints for choosing appropriate numbers of candidate vectors ($\delta_{\left(k\right)}^{k}$) for the calculation of observation-to-bit messages, the proposed QR-SBP detector can achieve acceptable error performance with smaller computational complexity compared to conventional standard BP and QR-BP detectors.
- After the end of the message updating procedures for the bit sequences in each transmit symbol, the hard-decision IC operation is performed using the estimated symbols. By applying the hard-decision IC operation, the size of the effective system model and corresponding bipartite graph decreases and the messages ($\alpha_{nm}$ and $\beta_{mn}$) for the next symbols can be updated without using the messages for the current symbol, leading to additional complexity reduction.
- The hard-decision IC operation in the proposed QR-SBP detector is enabled by performing a sequential bit-by-bit message updating procedure for each symbol. Although the messages for the current symbol are not directly utilized during the updating of the messages for the remaining symbols, they indirectly improve the quality of the messages for the remaining symbols by generating effective receive signal vectors using IC. Therefore, unlike conventional standard BP and QR-BP detectors with parallel processing structures, the messages for one symbol can update the messages for other symbols during each BP iteration, which can significantly accelerate convergence.

Based on these characteristics, the proposed QR-SBP detector with IC and candidate constraints can significantly improve convergence speed compared to conventional BP detectors while providing significantly reduced computational complexity for detection.

In the proposed scheme, because of employing the hard-decision IC, errors in the symbol detected earlier (e.g., ${\hat{x}}_{k}$) can be propagated to the remaining symbols (e.g., ${\hat{x}}_{k^{\prime }}$ with $k^{\prime }<k$), as in other detectors employing hard-decision IC [1,16]. The performance degradation from this error propagation can be minimized by employing the ordering of transmit symbols [1], e.g., sorted QR decomposition with a proper ordering criterion for the linear transformation [16,19].

Next, the computational complexity of the proposed QR-SBP detector is calculated and compared to those of the standard BP and QR-BP detectors. The computational complexity of the proposed QR-SBP detector is mainly governed by the initial linear transformation defined in (5) to (7), the IC operation in (11), and the calculation of $\beta_{mn}$ in (14) and (15). For the linear transformation, most of the complexity burden stems from the QR decomposition of the original channel matrix H, which requires a complexity of $O(MK^{2})$ [19]. For the hard-decision IC operation, *k* complex multiplications are required to obtain $z^{\left(k\right)}\left(={\tilde{y}}^{\left(k\right)}\left(1:k\right)\right)$, which entails a complexity of approximately O(K(K+1)/2) for each BP iteration. In addition, when considering both $X_{\left(k\right)}^{k-m+1,b}$ and b∈{0,1}, the number of candidate vectors for the calculation of $\beta_{mn}$ is at most $2\delta_{w}^{\left(k\right)}\cdot 2^{Q}$. Thus, because the number of $\beta_{mn}$ updated in each BP iteration is K(K+1)/2, the calculation of $\beta_{mn}$ in (14) and (15) requires a complexity of at most $O(K\left(K+1\right)\delta^{*}2^{Q})$, where $\delta^{*}=\max\delta_{w}^{\left(k\right)}\;\forall w,k$. Therefore, the upper bound of the computational complexity of the proposed QR-SBP detector after $t_{\max}$ iterations is approximately $O(MK^{2}+t_{\max}K\left(K+1\right)/2+t_{\max}K\left(K+1\right)\delta^{*}2^{Q})$, which is approximately proportional to δ and $K^{2}$ and exponentially increases with *Q*. In contrast, the computational complexities of the standard BP and QR-BP detectors are $O(t_{\max}MK2^{KQ})$ and $O(MK^{2}+t_{\max}\sum_{k=1}^{K}k2^{kQ})$, respectively, which increase exponentially with both *K* and *Q*. Therefore, the proposed QR-SBP detector requires much smaller computational complexity than conventional BP detectors, especially as *K* increases.

## 5. Simulation Results

In this section, the error performance of the proposed QR-SBP detector is evaluated. The average bit-error ratio (BER) and frame-error ratio (FER) are utilized as the error performance metrics of the detectors in uncoded and coded systems, respectively. To obtain reasonable average BER and FER, numerical simulations were performed until 1000 frame errors were counted for each signal-to-noise ratio (SNR) point, where each frame consists of 576 bits. In addition to the proposed QR-SBP detector, the linear LZF and LMMSE detectors as well as the standard BP and QR-BP detectors are considered as reference schemes. Furthermore, as an optimal bound for error performance, the ML detector and matched-filter bound (MFB) are considered for uncoded and coded systems, respectively, where the MFB is identical to the performance of the linear MF detector with K=1 and the same number of receive antennas *M* [1]. Quadrature phase shift keying modulation with Q=2 is also considered. In addition, the quasi-static fading channel in which the channel response is constant over a frame is considered. Further, the number of BP iterations for the BP-based detectors is set to eight. For the proposed scheme, a sorted QR decomposition with the ordering criterion to minimize the SNR of the symbol detected later (e.g., $x_{1}$) is utilized for the linear transformation [16]. Finally, $\delta_{\left(k\right)}^{w}$ for the proposed QR-SBP detector is set to two, regardless of *k* and *w*, unless otherwise specified.

### 5.1. Uncoded Systems

**Figure 2:**
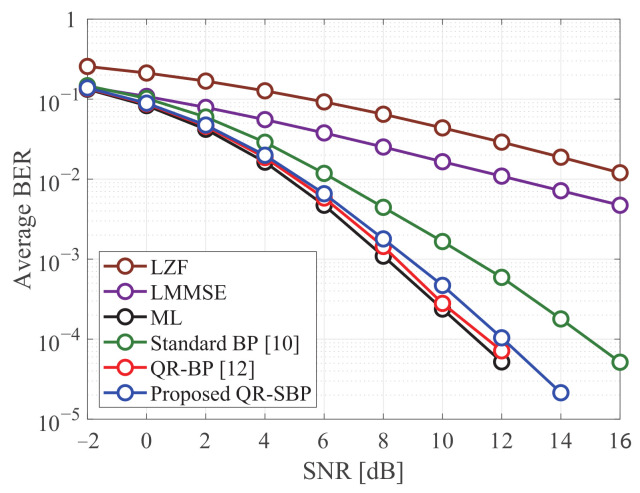
The average BERs of the detectors in uncoded 4 × 4 MIMO systems under Rayleigh fading.

**Figure 3:**
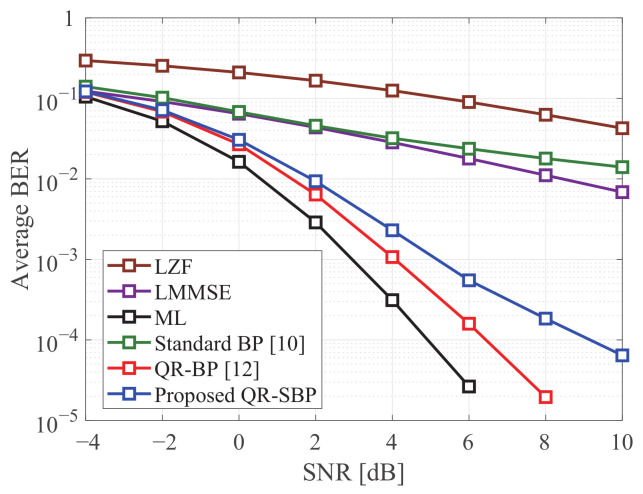
The average BERs of the detectors in uncoded 8 × 8 MIMO systems under Rayleigh fading.

Figure 2 and Figure 3 show the average BERs of the detectors in uncoded MIMO systems under Rayleigh fading when K=M=4 and K=M=8, respectively. It is shown that the proposed QR-SBP detector outperforms the standard BP and linear detectors on both antenna configurations. The average BER of the proposed QR-SBP detector is slightly degraded compared to those of the ML and QR-BP detectors, particularly when K=M=8. However, the SNR gains of the ML and QR-BP detectors over the proposed QR-SBP detector are marginal compared to that of the proposed QR-SBP detector over the standard BP and linear detectors. Because the proposed QR-SBP detector with $\delta_{\left(k\right)}^{w}=2$ requires much less computational complexity than the ML and QR-BP detectors, the proposed QR-SBP detector can be considered as an effective detection scheme for MIMO systems.

**Figure 4:**
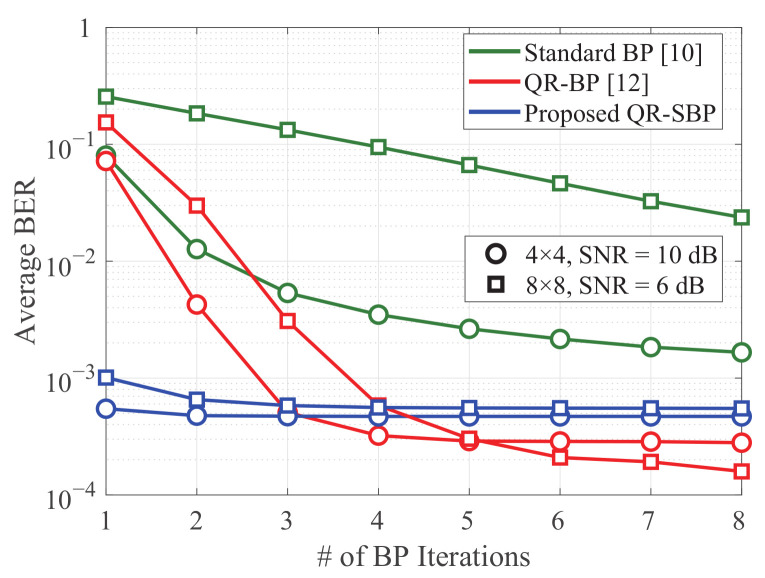
The average BERs according to the number of BP iterations in uncoded MIMO systems under Rayleigh fading.

In Figure 4, the average BERs of the BP detectors under Rayleigh fading are presented according to the number of BP iterations. Based on its serial updating procedure, the average BER performance of the proposed QR-SBP detector with one BP iteration is almost identical to the cases with more BP iterations. In other words, a small number of BP iterations can be sufficient for the convergence of the proposed QR-SBP detector in most scenarios. In addition, although the average BER of the QR-BP detector for higher numbers of BP iterations is better than that of the proposed QR-SBP detector, the performance of the QR-BP detector with a small number of BP iterations can be worse than that of the proposed QR-SBP detector, which demonstrates that the proposed QR-SBP detector requires a smaller number of BP iterations for convergence than the QR-BP detector. Furthermore, because of utilizing a fully connected bipartite graph, the standard BP detector exhibits the slowest convergence speed.

**Figure 5:**
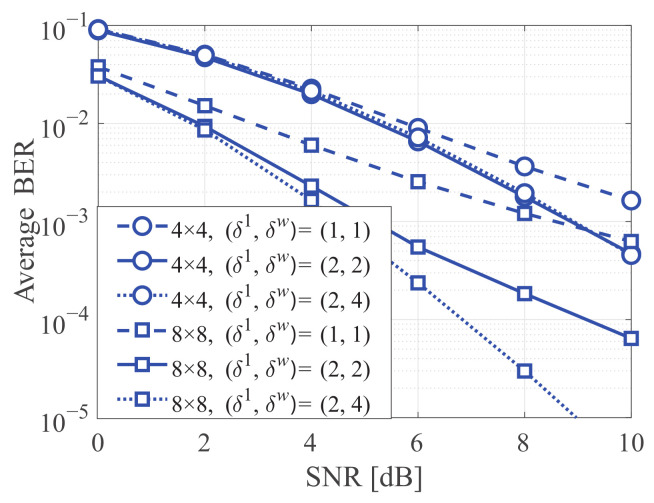
The average BERs according to the number of candidate vectors for the proposed QR-SBP scheme in uncoded MIMO systems under Rayleigh fading, where w≥2.

Figure 5 presents the average BERs of the proposed QR-SBP detector according to the number of candidates (i.e., $\delta_{\left(k\right)}^{w}$), where the Rayleigh fading channel is considered. Because $\delta_{\left(k\right)}^{w}$ for a given *w* is fixed, the subscript (k) is omitted for simplicity. It is clear that a larger $\delta^{w}$ can facilitate better error performance for the proposed QR-SBP detector at the cost of increased computational complexity. However, when K=M=4, the cases of $\left(\delta^{1},\delta^{w(\geq 2)}\right)=\left(2,2\right)$ exhibit nearly identical performance compared to the cases of $\left(\delta^{1},\delta^{w(\geq 2)}\right)=\left(2,4\right)$ (i.e., the maximum numbers of candidates for Q=2). This implies that the proposed QR-SBP detector can achieve acceptable error performance without using the maximum $\delta^{w}$ for a given system configuration. It is worth noting that even the proposed scheme with the maximum $\delta^{w}$ can provide significantly lower computational complexity than the standard BP and QR-BP detectors, as derived in Section 4.

**Figure 6:**
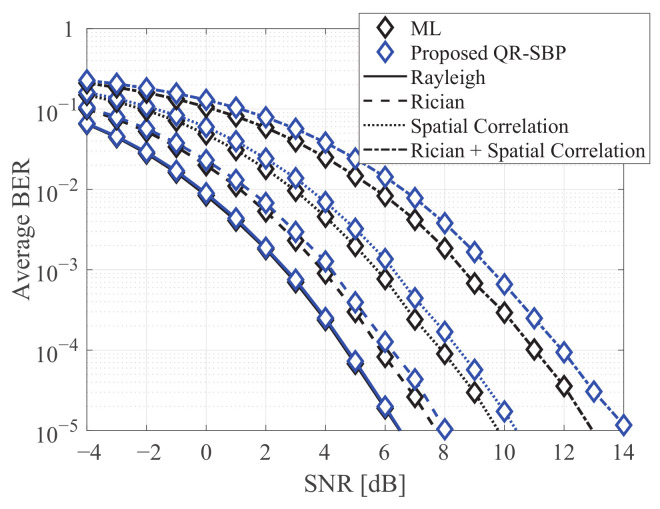
The average BERs of the ML and proposed QR-SBP detectors in uncoded 4 × 8 MIMO systems under various fading channels.

Figure 6 shows the average BERs of the ML and proposed QR-SBP detectors in uncoded MIMO systems under various fading channels, where K=4 and M=8. The Rician factor is set to 3 for the Rician fading channel, and the exponential correlation model with a coefficient of 0.5 is considered for both transmitter and receiver correlation matrices in the spatial correlation channel. For the Rayleigh fading channels, because of rich scattering, the proposed QR-SBP detector achieves the near-identical error performance to the ML detector. Meanwhile, as the channel correlation becomes severe, the average BERs of both detectors are degraded compared with those under Rayleigh fading because of the lack of the diversity gain, and the performance degradation of the proposed QR-SBP detector is relatively more significant than that of the ML detector. Nevertheless, the SNR gain of the ML detector over the proposed QR-SBP detector is marginal regardless of the fading channel, indicating that the proposed QR-SBP detector can achieve suboptimum error performance for various channel environments.

### 5.2. Coded Systems

In this subsection, the performance of the detectors is evaluated in MIMO systems with channel coding. A low-density parity-check code with a rate of 0.5 and codeword length of 576 is considered as a channel code. Multi-codeword transmission is considered (i.e., *K* codewords are generated and transmitted from each transmit antenna). For the BP detectors, iterative detection is considered [9] (i.e., the output LLR vector from each BP iteration is used as the input for the decoder and the output LLR vector from the decoder is used as the bit-to-observation message $\alpha_{nm}$ for the next BP iteration). For the proposed QR-SBP detector, the serial exchange of information between the detector and decoder is considered. In other words, following the calculation of the detector LLRs of the bits in a codeword (transmit antenna) during a BP iteration, the detector LLRs are used for decoding and the decoder LLRs are used for the IC operation in the BP iteration for the next codeword (transmit antenna). For the BP detectors, the number of decoding iterations for each codeword per BP iteration is set to 10, and thereby the total number of decoding iterations is 80. For the linear detectors and MFB, the total number of decoding iterations is also set to 80. In addition, an iteration stopping criterion is considered, i.e., the reception procedure of a codeword is over when the syndrome vector of the codeword after each decoding iteration is an all-zero vector.

**Figure 7:**
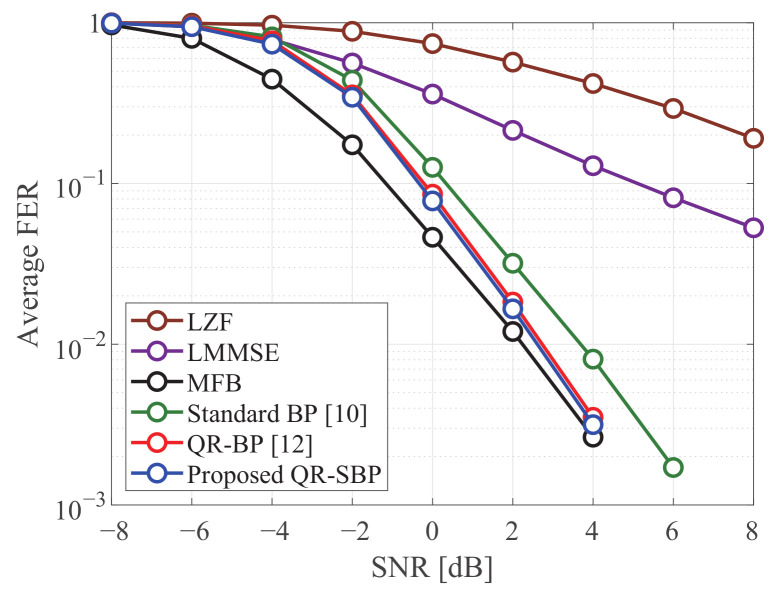
The average FERs of the detectors in coded 4 × 4 MIMO systems under Rayleigh fading.

**Figure 8:**
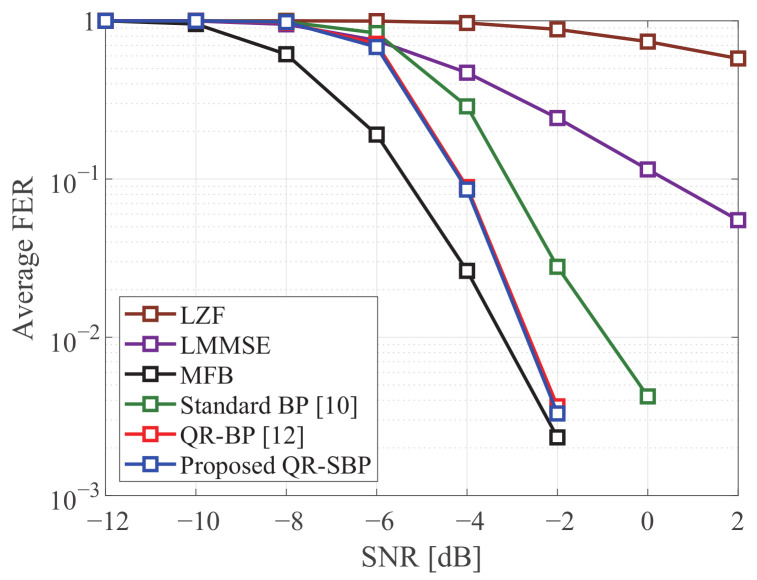
The average FERs of the detectors in coded 8 × 8 MIMO systems under Rayleigh fading.

Figure 7 and Figure 8 compare the average FERs of the detectors in coded MIMO systems under Rayleigh fading when K=M=4 and K=M=8, respectively. It is shown that the proposed QR-SBP detector can outperform the other schemes and achieve similar performance compared to the MFB and QR-BP detectors in both antenna configurations. Unlike the results for uncoded systems, the average FER of the proposed QR-SBP detector is nearly identical to that of the QR-BP detector for iterative detection when N=K=8. This shows that the proposed QR-SBP detector is also suitable for iterative detection, e.g., turbo equalization.

**Figure 9:**
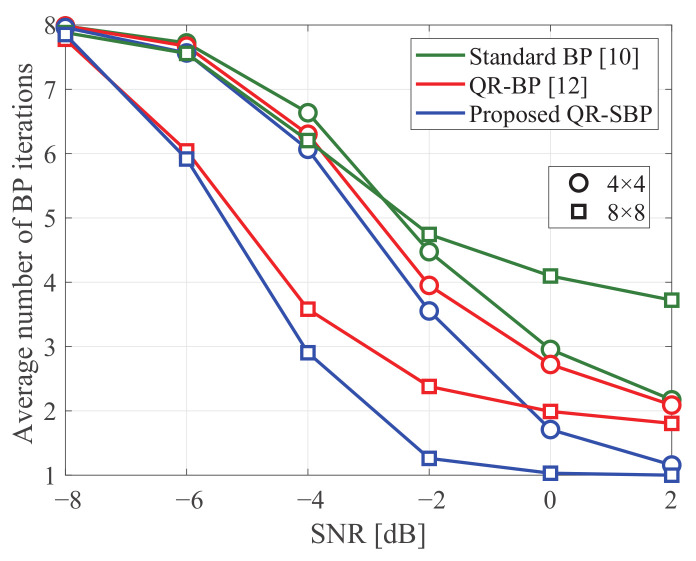
The average number of BP iterations in coded MIMO systems under Rayleigh fading.

Figure 9 shows the average numbers of BP iterations of the BP detectors for convergence in coded MIMO systems under Rayleigh fading when K=M=4 and K=M=8, where the average number of BP iterations for convergence is computed based on the BP iteration in which the iteration stopping criterion defined by the syndrome vector is satisfied. The standard and QR-BP detectors exhibit similar convergence speeds when K=M=4, and the QR-BP detector exhibits a faster convergence speed than the standard BP detector when K=M=8. Meanwhile, similar to the results in Figure 4 for uncoded systems, the proposed QR-SBP detector exhibits the fastest convergence speed, especially when K=M=8 in the high-SNR region. This verifies the effectiveness of the proposed QR-SBP detector in terms of convergence speed.

## 6. Conclusions

This paper proposed and investigated the QR-SBP detector for MIMO systems. The proposed QR-SBP detector utilizes IC and candidate constraints in a scheduled manner, which have not been considered in existing BP detectors for MIMO systems. By employing IC and candidate constraints in a scheduled manner, the proposed QR-SBP detector can provide suboptimum error performance with significantly reduced computation complexity and accelerated convergence speed compared to the conventional standard BP and QR-BP detectors. Specifically, the proposed QR-SBP detector has a complexity approximately proportional to the square of the number of transmit antennas, which is a huge reduction compared to the standard BP and QR-BP detectors with the complexity that increases exponentially with the number of transmit antennas. In addition, simulation results verify that the proposed QR-SBP detector can achieve suboptimum error performance with a small number of BP iterations, while the standard BP and QR-BP detectors require a number of BP iterations for convergence. Therefore, the proposed QR-SBP detector can be considered as an effective suboptimum detector for MIMO systems.

Throughout the paper, it is assumed that the proposed QR-SBP detector uses fixed numbers of candidate vectors for observation-to-bit message updating. This can be optimized according to the obtained messages and target system configuration. Furthermore, instead of the hard-decision IC operation, the soft-decision IC operation can be utilized to obtain a better error performance. In addition, in spite of the complexity reduction from the conventional BP detectors, the complexity of the proposed QR-SBP detector is still high for the cases of high-order modulation and large-scale MIMO systems, which needs to be reduced for such cases. These topics can be discussed in future works.

## Data Availability

Not applicable.

## Abbreviations

The following abbreviations are used in this manuscript:

MIMO	Multiple-input multiple-output
LZF	Linear zero-forcing
LMMSE	Linear minimum mean-square-error
ML	Maximum likelihood
MP	Message passing
BP	Belief propagation
QR-BP	QR-decomposition-based BP
IC	Interference cancellation
QR-SBP	Scheduled QR-BP with IC and candidate constraints
AWGN	Additive white Gaussian noise
LLR	Log-likelihood ratio
BER	Bit-error ratio
FER	Frame-error ratio
SNR	Signal-to-noise ratio
MFB	Matched-filter bound
**Mathematical Symbols**	
The following mathematical symbols are used in this manuscript:	
*K*	Number of transmit antennas
*M*	Number of receive antennas
*Q*	Number of bits assigned for each transmit symbol
*N*	Number of bits included in each transmit signal vector
X	$2^{Q}$-ary constellation set
y	M×1 receive signal vector
H	M×K MIMO channel matrix
$h_{m}$	*m*th row of H
x	K×1 transmit signal vector
n	M×1 AWGN vector
$b_{n}$	*n*th bit among the bit sequence in each x
$\alpha_{mn}$	Message from the *n*th bit node to the *m*th observation node
$\beta_{mn}$	Message from the *m*th observation node to the *n*th bit node
$X^{K,b}$	Set of K×1 vectors whose elements are the members of X with $b_{n}=0$ or 1
$l_{n}$	LLR of $b_{n}$
Q	M×M unitary matrix with $H=Q[R;0_{(M-K)\times K}]$
R	K×K upper triangular matrix with $H=Q[R;0_{(M-K)\times K}]$
$r_{m}$	*m*th row of R
${\tilde{r}}_{m}$	1×(K−m+1) vector containing the non-zero elements of $r_{m}$
$\tilde{y}$	K×1 receive signal vector for the QR-BP detector
$\tilde{n}$	K×1 AWGN vector for the QR-BP detector
${\hat{x}}_{k}$	A hard decision symbol of $x_{k}$
${\tilde{y}}^{\left(k\right)}$	K×1 receive signal vector for the message updating of the bit sequence in $x_{k}$
$z^{\left(k\right)}$	k×1 effective receive signal vector for $x_{k}$ in the proposed QR-SBP detector
$x^{\left(k\right)}$	k×1 effective transmit signal vector for $x_{k}$ in the proposed QR-SBP detector
${\tilde{n}}^{\left(k\right)}$	k×1 effective AWGN vector for $x_{k}$ in the proposed QR-SBP detector
$R^{\left(k\right)}$	k×k effective channel matrix for $x_{k}$ in the proposed QR-SBP detector
$B_{mn}^{b}$	Set to update $\beta_{mn}$ with b=0 and 1 in the proposed QR-SBP detector
${{\tilde{r}}_{m}}^{\left(k\right)}$	1×w vector containing the non-zero elements of the *m*th row of $R^{\left(k\right)}$
$X_{\left(k\right)}^{w,b}$	Set of w×1 vectors for $x_{k}$ whose elements are the members of X with $b_{n}=b$
$\delta_{\left(k\right)}^{w}$	Number of surviving w×1 vectors in $X_{\left(k\right)}^{w,b}$
$Y_{\left(k\right)}^{w,b}$	Set containing the $\delta_{\left(k\right)}^{w}$ surviving w×1 vectors in $X_{\left(k\right)}^{w,b}$
$\delta^{*}$	Maximum $\delta_{w}^{\left(k\right)}$ for all *w* and *k*
$t_{\max}$	Number of maximum BP iterations
